# Phenology of a Vegetation Barrier and Resulting Impacts on Near-Highway Particle Number and Black Carbon Concentrations on a School Campus

**DOI:** 10.3390/ijerph14020160

**Published:** 2017-02-08

**Authors:** Christina H. Fuller, David R. Carter, Matthew J. Hayat, Richard Baldauf, Rebecca Watts Hull

**Affiliations:** 1 Division of Environmental Health, Georgia State University School of Public Health, P.O. Box 3995, Atlanta, GA 30302, USA; 2 Division of Epidemiology and Biostatistics, Georgia State University School of Public Health, P.O. Box 3995, Atlanta, GA 30302, USA; rcar371@gmail.com (D.R.C.); mhayat@gsu.edu (M.J.H.); 3 Office of Research and Development, U.S. Environmental Protection Agency, Mail Drop 8101R, Research Triangle Park, NC 27711, USA; Baldauf.Richard@epa.gov; 4 School of History and Sociology, Georgia Institute of Technology, Atlanta, GA 30332, USA; rwattshull@gatech.edu

**Keywords:** black carbon, particulate matter, vegetation, barrier, highway, near-road, season

## Abstract

Traffic-related air pollution is a persistent concern especially in urban areas where populations live in close proximity to roadways. Innovative solutions are needed to minimize human exposure and the installation of vegetative barriers shows potential as a method to reduce near-road concentrations. This study investigates the impact of an existing stand of deciduous and evergreen trees on near-road total particle number (PNC) and black carbon (BC) concentrations across three seasons. Measurements were taken during spring, fall and winter on the campus of a middle school in the Atlanta (GA, USA) area at distances of 10 m and 50 m from a major interstate highway. We identified consistent decreases in BC concentrations, but not for PNC, with increased distance from the highway. In multivariable models, hour of day, downwind conditions, distance to highway, temperature and relative humidity significantly predicted pollutant concentrations. The magnitude of effect of these variables differed by season, however, we were not able to show a definitive impact of the vegetative barrier on near-road concentrations. More detailed studies are necessary to further examine the specific configurations and scenarios that may produce pollutant and exposure reductions.

## 1. Introduction

Air quality is a pressing issue in the urban environment contributing significantly to the global burden of disease [1]. Traffic-related air pollution (TRAP) is a mix of chemicals produced from the burning of diesel or gasoline in vehicles. TRAP includes particulate matter (PM), oxides of nitrogen, carbon monoxide, carbon dioxide, polycyclic aromatic hydrocarbons (PAHs) and metals as well as particulate emissions from brake and tire wear. TRAP has been associated with adverse health outcomes including asthma, lung cancer, cardiovascular disease and mortality [2]. A 2010 assessment by the Health Effects Institute that evaluated multiple epidemiological studies concluded that there was “sufficient” evidence for long-term exposure to TRAP and asthma exacerbation in children and borderline “sufficient” evidence for asthma onset in children. The report concluded that there was “suggestive, but not sufficient” evidence for exposure to TRAP and all-cause mortality, cardiovascular morbidity and decrements in lung function for children and adults [2].

Unlike regional air pollutants there is a high degree of spatial variation of TRAP. Roadway layout, traffic volume and fleet mix drive the concentration of TRAP near roads and highways [3]. Some components exhibit steep gradients near roads particularly carbon monoxide, nitric oxide, nitrogen dioxide, black carbon and ultrafine (diameter <0.1 µm) particles (UFP) [3,4]. Near road concentrations are related not only to roadway proximity, but also local meteorology, including wind direction, wind speed and precipitation [3]. Higher concentrations are typically found when measured downwind of roadways compared to upwind sites. Concentrations also vary by height, usually decreasing with increasing distance from the ground. High winds increase turbulence and dispersion and tend to decrease concentrations, while very calm or no wind may increase local concentrations [3,4].

Health effects have been most closely linked to the particulate matter component of TRAP including respiratory conditions, cardiovascular disease and lung cancer [5]. Field studies identify the highest exposures to TRAP to be for those traveling in traffic and living close to major roads and highways [3,6,7,8]. Multiple methods have been proposed and implemented to reduce air pollutant concentrations and population exposures to air pollutants on and near large roads and highways including motor vehicle tailpipe emission standards, increased mass transit to reduce vehicle activity, limiting time spent near large roads and installing solid physical barriers [9,10]. Given the cost and complexity of these strategies, municipalities and communities may be interested in identifying other options to reduce the impact of local sources of air pollution. One possibility gaining attention is the use of vegetation to reduce concentrations of TRAP at both the urban (city) and micro (street) scales [11,12,13,14,15].

In general, TRAP concentrations are higher in urban areas; which usually corresponds to lower vegetative cover although there is significant variation in vegetative cover between cities [12]. Nowak et al., created a model comparing the number of trees, tree density, tree cover, leaf area index (LAI), and most common tree species for 14 U.S. cities [16]. The lowest model estimates were recorded in Casper (WY, USA) for tree density (9.1 trees/ac), tree cover (8.9%), and LAI (0.3), and the highest measurements in Atlanta (GA, USA) for tree density (111.6 trees/ac), tree cover (36.7%), and LAI (2.2). Estimates for increased PM_2.5_ removal and air quality improvements were related to greater tree density, cover, and LAI and the total amount of PM_2.5_ removal annually by trees varied from 4.7 tonnes in Syracuse (NY, USA) to 64.5 tonnes in Atlanta (GA, USA) [13]. Removal of up to 0.36 g·m^−2^·year^−1^ was estimated for Atlanta (GA, USA) [13]. Average annual percent air quality improvement ranged between 0.05% in San Francisco (CA, USA) to 0.24% in Atlanta (GA, USA). Pollutant removal is a function of the absolute concentration in the air, which makes it difficult to draw accurate conclusions between studies due to differences in pollution levels between the cities sampled [13,17,18].

Separate reviews have summarized the effect of vegetation on near-roadway environmental quality [11,14,15,19,20]. Each concluded that vegetation could be effective in improving air quality when designed appropriately for local conditions [11,14,15,19]. However, under specific circumstances, such as street canyons, vegetation has been found to increase pollutant concentrations in both experimental and field studies [9,21,22]. Roadside vegetation is able to reduce ambient PM concentrations by increasing dispersion, interfering in pollutant transport and direct interception of particles [11,20]. Studies have shown that effectiveness may differ based on particle size [20,23]. Some particles adsorb onto the leaf surface, while others penetrate into the wax layer. Adsorbed particles can be resuspended by wind or washed off by rain and deposited onto the ground causing chemicals in soil to build up over time [24,25]. Therefore, understanding particular sources is key to the construction of effective barriers. 

We sought to expand upon past work by examining a vegetative barrier at multiple time points. This study evaluated the phenology of a vegetation barrier and its resulting impact on near-highway particle number and black carbon concentrations on a school campus.

## 2. Materials and Methods

### 2.1. Study Design

**Figure 1:**
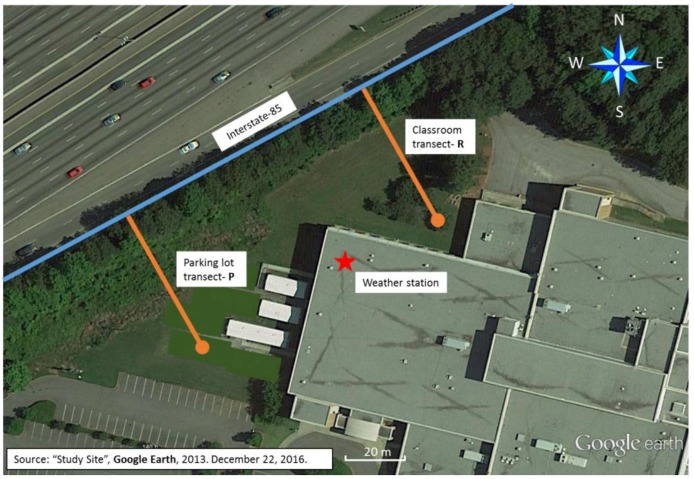
Study site and monitoring transects.

The study took place at a middle school in Georgia adjacent to Interstate-85 (I-85), which carries approximately 150,000 vehicles per day [26]. The school was selected based on its proximity to the highway and an existing barrier of evergreen and deciduous trees approximately 200 meters (m) long and 12 m tall. Measurements were conducted at a distance of 10 m and 50 m from the highway along two transects labeled R (in the direction of an outdoor classroom) and P (in the direction of the parking lot) (Figure 1). PNC (particle number concentration) data was collected using a handheld alcohol-based condensation particle counter (CPC Model 3007, TSI Inc., Shoreview, MN, USA). This instrument measures the total particle number concentration for all particles between 7 nm and 3 µm in diameter. Black carbon is a class of carbonaceous particles and was measured using a microaetholometer (MicroAeth, Model AE51, AethLabs, San Francisco, CA, USA) at a flow rate of 100 mL·min^−1^. The MicroAeth measures rate of change in absorption of transmitted 880 nm wavelength light through a filter where the aerosol is deposited. PNC data were collected as 1-min averages and BC (black carbon) data as 5-min averages. BC data were collected at this resolution to improve the stability of the reading [27]. Two instruments of each type were run side-by-side in the lab on multiple days and also approximately 10 min at the start and conclusion of each monitoring session in order to evaluate quality control.

Meteorology data were collected onsite using a mobile weather station (Vantage Pro2, Davis Instruments, Hayward, CA, USA). The weather station was located on the roof of the school building at approximately 11 m above ground level. The site was unobstructed and between the two transects. Data were collected at 1-min intervals concurrent with pollutant measurements for wind speed, wind direction, temperature and relative humidity. Hourly traffic data for I-85 were collected from a permanent station near the school [26]. The data were downloaded from a public website run by the Atlanta Regional Commission. Total traffic count was calculated based on the sum of vehicles travelling in the northbound and southbound directions.

### 2.2. Vegetation and Calculation of Leaf Area Index

**Figure 2:**
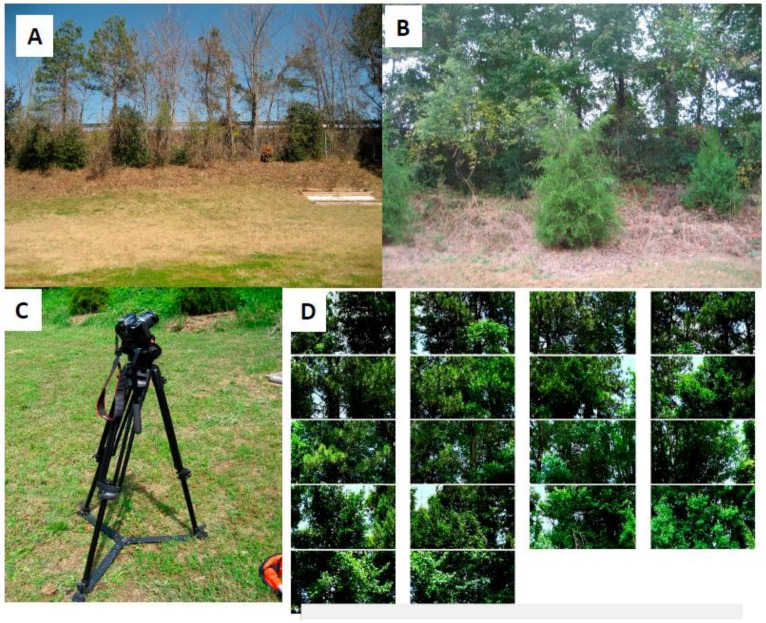
(Panel **A**) shows a section of the vegetative barrier during a time of lower leaf area index (LAI); (Panel **B**) shows the vegetative barrier during a time of higher LAI; (Panel **C**) shows the camera set up to take photos used in LAI calculation; (Panel **D**) shows an example of the photos used to calculate LAI.

The vegetative barrier was comprised of a mixture of evergreen and deciduous trees (Figure 2A,B). Foliage was estimated by the leaf area index (LAI), defined as the total one-sided green leaf area per unit of ground surface [28]. We selected an indirect optical method using CAN-EYE software (INRA-UAPV, Avignon, France) to process images to estimate LAI. CAN-EYE software was used because it is capable of processing digital cover images with a standard point and shoot digital camera.

Digital images were taken using both single-lens-reflex (DSLR) and point-and-shoot cameras mounted on a tripod (Figure 2C). Two cameras, a Canon Powershot SD630 sensor type 1/2.5″ CCD width 5.76 mm (Canon, Tokyo, Japan) and Canon EOS Rebel T2i/550D sensor type CMOS width 14.9 were used for comparison. The tree line was used as the point of reference for the images. Images were taken 4 m from the tree line with the camera inclined at 57.5° angle from the vertical (Figure 2C). The images were constrained to the same format, size, camera set-up, and direction to meet image software-processing requirements. Images were downloaded and given proper labels to organize the images spatially along the tree line. Calculations of LAI were made by integrating photographs into CAN-EYE software (Figure 2D). Both cameras produced similar estimates for LAI, therefore, we selected the estimates based on the Canon EOS Rebel T2i/550D.

## 3. Results

We collected data over 15 days between 12 March 2013 and 7 February 2014 during the three campaigns in spring (March, April and May), fall (November) and winter (January and February). Monitoring periods were carried out between 8 a.m. (8 h) and 2 p.m. (14 h) and each session lasted between 4 and 6 h. BC readings underwent post-processing using a smoothing algorithm according to the method used by Hagler et al. [27]. Due to malfunctioning of the instrumentation we do not have valid PNC data from January or February 2014. A total of 4204 PNC measurements at 1-min resolution and 1216 black carbon measurements at 5-min resolution were collected (Table 1).

Comparison of data from quality control monitoring in the laboratory and in the field show good correlation between pairs of identical instruments when collecting measurements side-by-side. The correlation between instruments was 0.98 for PNC and the mean difference for measurements was 526 particles·cm^−3^ (SD: 3035 particles·cm^−3^). Measurements for BC had a correlation of 0.94 and the mean difference was 0.24 µg·m^−3^ (SD: 2.11 µg·m^−3^).

### 3.1. Meteorology, Traffic and Leaf Area Index

**Figure 3:**
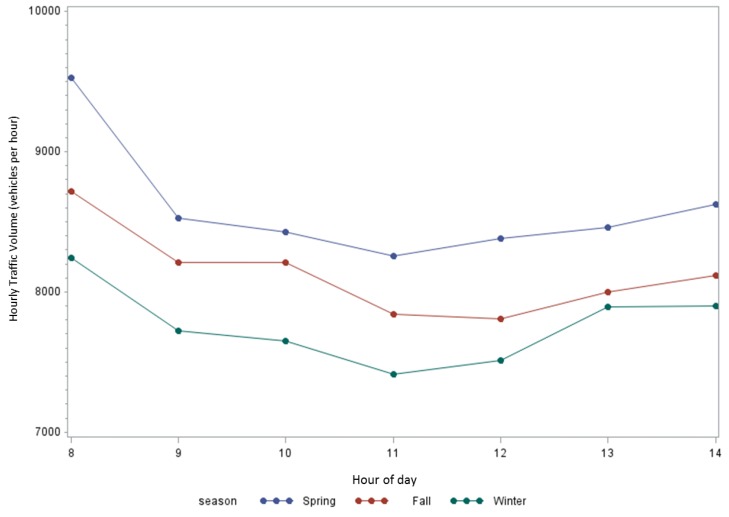
Hourly traffic volume on Interstate-85 separated by season.

Descriptive statistics of meteorological parameters and traffic volume are given in Table 2 and a graph of traffic volume is presented in Figure 3. Traffic volume differed by season with a mean hourly flow of 8684 vehicles per hour in the spring, 8271 in the fall and 7881 in the winter. Traffic volume was highest in the morning with approximately 9528 vehicles per hour in spring, 8718 in fall and 8241 in winter. Traffic volume generally decreased from the morning through early afternoon. The mean temperature was 14.8 °C (SD: 10.8) ranging from −6.4 °C to 31.6 °C. In order to explore the impact of wind direction on pollutant concentrations we identified downwind and upwind conditions. Downwind conditions occur when the wind blows across the highway toward the study site, while upwind conditions occur when the opposite is true. Directions that create downwind conditions were determined based on an aerial photo of the site and included six wind directions (W, WNW, NW, NNW, N, NNE AND NE). The remaining wind directions were categorized as upwind. Across all monitoring periods, approximately 46 percent of the monitoring periods took place under downwind conditions with the remaining 54 percent being upwind conditions. The percentage of time during monitoring under downwind conditions differed between seasons and was substantially higher on the monitoring days in winter. LAI was calculated as 3.6 in May, 1.8 in November and 2.5 in January/February. LAI measurement was not taken at the start of the first sampling campaign in March. Our visual records reported that the most tree foliage was present in May, which decreased in November and further in January. Therefore, it was surprising that LAI estimates were higher in January/February than November. After examination of the original photos it was determined that this was likely the result of the photos being taken on sunny days in May and November, while the photos in January were taken on a cloudy day. The cloudy background was interpreted by the software as foliage thereby resulting in a higher estimate for LAI.

### 3.2. Pollutant Concentrations

**Figure 4:**
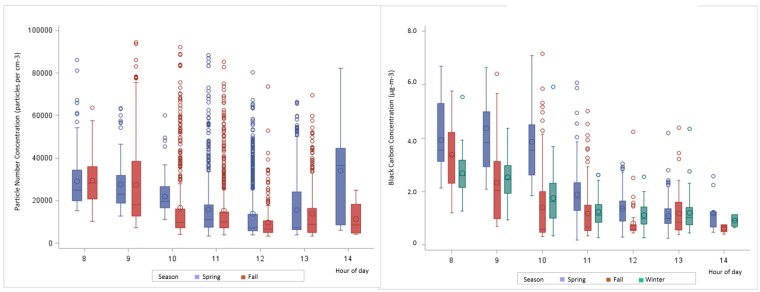
Pollutant concentrations according to hour of day and season.

We report PNC as 1-min averages and BC as 5-min averages here and for the remainder of the paper unless noted otherwise. Both PNC and BC data were highly skewed as can be seen by comparing mean and median values given in Table 3. Spearman rank correlation between the two pollutants, averaged over 5 min, showed a strong association with a correlation of 0.77 (*p* < 0.001) (data not shown). The classroom and parking lot transects were combined for analysis to increase the sample size and also because the LAI calculation was based on the vegetative barrier incorporating both transects. Pooling all data, the highest PNC was measured in the fall. Mean PNC decreased between 10 m and 50 m approximately 32% across all time periods, however, the decrease was much higher in fall compared to spring. The same was true for median concentrations in the fall, however, the median PNC was higher at 50 m compared to 10 m in the spring. The highest concentrations of BC were found in the spring. Mean BC concentrations were reduced by approximately 31% between 10 m and 50 m across all seasons with a similar trend for median concentrations. When examining the effect of time, PNC decreased in magnitude between 8 and 13 h with the exception of an increase at 14 h in the spring (Figure 4). Both the range and level of BC concentrations decreased between 8 h and 14 h over all seasons. The mean ratio of BC concentrations between 10 m and 50 m was 1.67 in winter, 1.40 in fall and 1.24 in spring. The mean ratio for PNC was 2.06 in fall and 1.02 in spring.

### 3.3. Multivariable Models

Next, we built models for each season that describe 1-min averaged PNC and 5-min averaged BC (See Table 4). PNC and BC were not normally distributed therefore we log-transformed the concentrations and used the transformed values in linear mixed models. Mixed models allow for dependency between pollutant measurements observed at repeated points in time. Based on criteria of statistical significance and information criteria, we selected a compound symmetry correlation structure [29]. We included traffic volume, distance, hour of day and meteorological parameters in all models a priori. (We excluded dew point due to high correlation with another included variable).

In multivariable models significant predictors of PNC for spring and fall included hour of day, downwind conditions and relative humidity for both spring and fall time periods. Each hour change between 8 h and 14 h was associated with a 15% (spring) or 22% (fall) decrease in concentrations. Downwind conditions increased concentrations by 71% (spring) and 106% (fall). In spring wind speed, temperature and traffic volume were also significant. A change in distance from 50 m to 10 m was significant only in fall.

Multivariable models of BC were similar in that hour of day, downwind conditions and distance were significant across all time periods. Each additional hour between 8 h and 14 h was associated with reductions in concentrations between 11% and 30%, depending on the season. The effect of downwind conditions also varied by season, increasing concentrations from 47% to 160%. The change in distance from 50 m to 10 m was associated with an 8% to 59% increase in BC concentrations. Traffic volume was significant only in the spring while temperature and relative humidity were significant in both spring and winter.

## 4. Discussion

We evaluated levels of particle number concentration (PNC) and black carbon (BC) at a school next to a major highway with a vegetative barrier that varied in leaf foliage over time. Although we did not measure the levels of PNC and BC on the roadway itself, our results provide insight into the pollutant concentration changes that occurred in close proximity to the road. Due to inconsistency in the application of the methodology for measurement of LAI, we are confident in the estimates made for spring and fall only. These values show a decrease in LAI between the two time periods, which correlates with field notes reporting the most foliage being present in spring, less in fall and the least in winter. Therefore, season, within the scope of this study, is a reasonable proxy for LAI.

We identified consistent decreases in mean and median BC measured at 50 m compared to 10 m distance from the highway. The ratios comparing 10 m BC concentrations to 50 m concentrations were highest for winter, followed by fall and then spring. It is possible that less foliage of the vegetative barrier in winter allows for pollution to be transported from the highway onto the school property being affected by expected dispersion and dilution processes. However, more foliage in fall and spring could enhance dispersion and mixing reducing concentrations at 10 m and making a gradient by distance less pronounced. Other studies have also noted more gradual reductions in concentration of black carbon and UFP behind vegetative barriers as well as differences in collection efficiency [23,30,31]. Compared to BC concentrations, there was greater variation in PNC between spring and fall monitoring periods. Reductions in concentrations between 10 m and 50 m were evident for mean, but not median, concentrations. This may be due to the existence of other sources of PNC that remain unmeasured and may have more of an impact depending on local conditions. Local conditions include wind direction and speed, which differed between the two periods. Another study found both increases and decreases in concentrations next to vegetative barriers depending on the spatial layout as well as local weather conditions [25]. In addition, the importance of particle contributions from the highway may be complicated, because the two transects were relatively close to corners of the building. Therefore, the monitoring locations may have been affected by increased turbulence and corner eddies, which would in turn impact concentrations. The location of the meteorological monitoring station being on the rooftop would not have been able to capture these small local wind fields. Lastly, the particular structure of the vegetative barrier in this study is non-uniform resulting in various porosity and collection efficiencies [30].

We sought to evaluate individual predictors while controlling for others using multivariable models. Due to the limited number of estimates we were unable to include LAI in these models. However, we did build separate models by season that we can evaluate with the background knowledge of vegetative foliage during each time period. There was a consistent effect of hour of day on both BC and PNC. Pollutant concentrations and traffic volume were highest earlier in the morning and decreased through the early afternoon. Traffic volume is the proposed source of pollutant concentrations. Although the estimated effects of higher traffic volume were increased pollutant concentrations they were only statistically significant in spring, when traffic flow was the highest. The lack of statistical significance may be due to insufficient variability within the sampling period. Another likely contributor to the reduction in concentrations during this time frame is the rise of the atmospheric boundary layer as well as increases in atmospheric instability resulting in more dispersion and a reduction in concentrations. We also evaluated wind direction by categorizing it into downwind and upwind conditions. Downwind conditions would increase concentrations by transporting pollution from the proposed source, the highway, toward the monitoring instruments. Wind direction varied substantially by season with the lowest percentage of downwind conditions occurring in the spring at 26%, 32% in the fall and 79% in the winter. In the models, downwind conditions increased PNC by a greater amount in fall compared to spring. This may be an effect of the vegetative barrier whereby higher foliage in spring increases dispersion and dampens the effect of downwind conditions compared to fall. However, the effect of downwind conditions does not show this same pattern with BC. The effect of distance to highway was consistently associated with higher BC concentrations at 10 m. The magnitude of the effect increased from an 8% increase in spring to a 59% increase in winter. This may show an effect of the vegetative barrier given that other variables common to season and pollution, such as temperature, were controlled for in the model. 

Our study has limitations based on its design as well as unforeseen circumstances. We have only two valid measurements of LAI, which is not sufficient to include in statistical analyses, however, we did take this information into account when interpreting pollutant concentrations. Another limitation of the dataset was the use of two distances to represent a gradient in pollutant concentration. Measurements on the highway side of the barrier and at additional distances beyond the barrier would allow for a detailed assessment of a gradient better than a simple ratio. However, since the school is in close proximity to the roadway the measurements are representative of levels directly outside the school building in an area that is used by students and staff. The vegetative barrier exists naturally and there is a high degree of variation in thickness, tree species and coverage along its length. Some portions of the barrier may act as a filter to pollutants while other sections may not. This heterogeneity would have added some degree of error into our LAI estimates, however, the methodology that we used is appropriate for a barrier of this type. Another limitation is the lack of data on atmospheric stability and mixing height to include in our assessment. The addition of this data could help to interpret the concentrations that were measured and explain additional variation in statistical models.

These results have practical importance given the levels of PNC and BC concentrations measured in an area that is used as an outdoor classroom. Therefore, there is potential for pollutant exposure for the children if using that area. The U.S. Environmental Protection Agency (EPA) 2011 School Siting Guidelines recommend evaluation of air pollution concentrations for any sites under consideration located within a half-mile of a high traffic road or highway [32]. For schools close to highways or busy roadways that cannot feasibly be relocated, exploring options to reduce particulate matter concentrations on school grounds may be of high priority for administrators. Based on the design of this study we are not able to definitely show a benefit of the existing vegetation at this site, but the results suggest that further study is warranted.

## 5. Conclusions

Over the span of 3 seasons we measured the phenology of a vegetative barrier and levels of PNC and BC out to a distance of 50 m from a major interstate highway. Field records note that foliage of the vegetative barrier was highest in spring and lowest in winter, which is supported by LAI calculations. We identified consistent decreases in BC concentrations with increased distance, however, PNC was more complex. The presence of other sources of PNC or unmeasured factors may explain these results. Multivariable models explaining BC and PNC noted differences in effect estimates based on season. Distance to highway, hour of day, downwind conditions, temperature and relative humidity significantly predicted pollutant concentrations in the majority of models. A statistically significant effect of traffic volume on an increase in concentrations was seen only in the spring. The effect of distance and wind direction may be explained by the existence of the vegetative barrier. However, we were not able to show a definitive impact of the vegetative barrier on near-road concentrations based on the restrictions of the study design. Given the need to identify options for pollution mitigation for near-highway communities, more detailed studies are advised to thoroughly examine the ability for vegetative barriers to reduce near-roadway pollution.

## Figures and Tables

**Table 1:** Number of measurements collected during the study period.

Season	PNC	BC
	(1-Min Averages)	(5-Min Averages)
**Spring**	2370	472
**Fall**	1834	367
**Winter**	0	377

PNC = particle number concentration, BC = black carbon.

**Table 2:** Summary statistics of meteorology and traffic during the monitoring periods.

Parameter	Spring						Fall						Winter					
	Mean (SD)	Min	25%	Median	75%	Max	Mean (SD)	Min	25%	Median	75%	Max	Mean (SD)	Min	25%	Median	75%	Max
Traffic Volume (vehicles per h)	8684 (787)	7590	8037	8478	9244	11,073	8271 (597)	7633	7887	8071	8,441	9988	7881 (529)	7269	7431	7748	8207	9159
Temperature (°C)	25.9 (3.2)	19.9	23.6	25.7	28.7	31.6	14.7 (3.7)	5.5	12.3	14.9	16.9	21.7	2.6 (4.5)	−6.4	−0.9	2.3	7.0	9.8
Relative Humidity (%)	59.8 (13.0)	40.0	51.0	57.0	63.0	96.0	56.6 (15.8)	34.0	42.0	55.0	70.0	89.0	53.9 (18.2)	20.0	42.0	51.0	69.0	89.0
Dewpoint (°C)	17.2 (2.9)	10.9	15.5	18.4	19.6	21.1	5.7 (5.3)	−1.4	2.0	3.5	12.0	14.0	−6.6 (6.8)	−20.1	−16.7	−3.5	−1.8	0.1
Wind speed (m/s)	1.5 (0.8)	0.0	0.9	1.3	1.8	4.9	1.6 (0.7)	0.0	0.9	1.8	2.2	4.5	2.2 (1.4)	0.0	1.3	1.8	2.7	8.5
Wind direction (%)																		
Downwind	26%						32%						79%					
Upwind	74%						68%						21%					

SD = Standard deviation.

**Table 3:** Near-highway measurements of particle number and black carbon concentrations alongside the highway and vegetative barrier.

	Spring				Fall				Winter			
PNC * (particles·cm^−3^)	Mean	SD	Median	IQR	Mean	SD	Median	IQR	Mean	SD	Median	IQR
All data	16,934	14,716	10,089	17,352	17,719	16,592	11,177	14,257	-	-	-	-
10 m	17,455	16,128	9039	19,145	24,356	20,480	15,655	28,008	-	-	-	-
50 m	16,411	13,134	11,190	14,637	11,097	6623	9519	7781	-	-	-	-
Difference (10 m–50 m)	361	9394	−361	3945	13,221	16,614	5646	18,876	-	-	-	-
Ratio (10 m/50 m)	1.02	0.36	0.95	0.30	2.06	1.09	1.56	1.24	-	-	-	-
Black carbon ** (µg·m^−3^)												
All data	1.81	1.41	1.45	1.33	1.54	1.41	0.79	1.73	1.65	0.91	1.41	1.29
10 m	2.09	1.76	1.57	1.54	1.82	1.63	0.96	2.40	1.99	0.94	1.76	1.30
50 m	1.52	0.85	1.35	1.12	1.26	1.08	0.72	1.24	1.30	0.72	1.05	1.04
Difference (10 m–50 m)	0.55	1.11	0.09	1.06	0.60	0.81	0.26	0.81	0.69	0.49	0.64	0.53
Ratio (10 m/50 m)	1.24	0.46	1.10	0.74	1.40	0.43	1.33	0.57	1.67	0.52	1.57	0.59

PNC = particle number concentration, SD = Standard Deviation, IQR-Interquartile range; * 1-min averages; ** 5-min averages.

**Table 4:** Multivariable models of near-highway log-transformed particle number concentration (PNC) and black carbon (BC) concentration.

**PNC**	**Spring**				**Fall**				**Winter**			
**Effect**	**Estimated Effect**	**Lower 95% CI**	**Upper 95% CI**	***p*-Value**	**Estimated Effect**	**Lower 95% CI**	**Upper 95% CI**	***p*-Value**	**Estimated Effect**	**Lower 95% CI**	**Upper 95% CI**	***p*-Value**
Traffic Volume (per 1000 vehicles·h^−1^)	1.16	1.12	1.21	<0.01	1.16	0.99	1.35	0.07	-	-	-	-
Distance (=10 m)	1.01	0.97	1.04	0.72	1.79	1.70	1.88	<0.01	-	-	-	-
Hour of day	0.85	0.82	0.88	<0.01	0.78	0.74	0.83	<0.01	-	-	-	-
Downwind Conditions	1.71	1.63	1.79	<0.01	2.06	1.95	2.18	<0.01	-	-	-	-
Wind Speed (m/s)	1.06	1.03	1.09	<0.01	0.96	0.92	1.00	0.03	-	-	-	-
Temperature (°C)	1.05	1.04	1.06	<0.01	0.99	0.97	1.01	0.33	-	-	-	-
Relative Humidity (%)	1.02	1.01	1.02	<0.01	0.99	0.99	0.99	<0.01	-	-	-	-
**BC**	**Spring**				**Fall**				**Winter**			
**Effect**	**Estimated Effect**	**Lower 95% CI**	**Upper 95% CI**	***p*-Value**	**Estimated Effect**	**Lower 95% CI**	**Upper 95% CI**	***p*-Value**	**Estimated Effect**	**Lower 95% CI**	**Upper 95% CI**	***p*-Value**
Traffic Volume (per 1000 vehicles·h^−1^)	1.34	1.23	1.45	<0.01	1.05	0.68	1.60	0.82	1.12	0.96	1.31	0.16
Distance (=10 m)	1.08	1.00	1.16	0.05	1.27	1.11	1.46	<0.01	1.59	1.48	1.71	<0.01
Hour of day	0.75	0.69	0.82	<0.01	0.70	0.61	0.81	<0.01	0.89	0.84	0.95	<0.01
Downwind Conditions	1.72	1.55	1.92	<0.01	2.60	2.23	3.04	<0.01	1.47	1.33	1.62	<0.01
Wind Speed (m/s)	0.98	0.91	1.05	0.49	0.95	0.82	1.10	0.51	1.00	0.96	1.05	0.94
Temperature (°C)	1.14	1.12	1.16	<0.01	1.03	0.97	1.10	0.27	0.98	0.97	1.00	0.01
Relative Humidity (%)	1.02	1.01	1.02	<0.01	1.00	0.99	1.00	0.43	1.01	1.00	1.01	0.02

CI = Confidence interval.
